# Consumers’ Responses to Front-of-Pack Nutrition Labelling: Results from a Sample from The Netherlands

**DOI:** 10.3390/nu11081817

**Published:** 2019-08-06

**Authors:** Manon Egnell, Zenobia Talati, Marion Gombaud, Pilar Galan, Serge Hercberg, Simone Pettigrew, Chantal Julia

**Affiliations:** 1 Nutritional Epidemiology Research Team (EREN), Sorbonne Paris Cité Epidemiology and Statistics Research Center (CRESS), U1153 Inserm, U1125 Inra, Cnam, Paris 13 University, 93000 Bobigny, France; 2 School of Psychology, Curtin University, Kent St, Bentley, WA 6102, Australia; 3 Public Health Department, Avicenne Hospital, AP-HP, 93000 Bobigny, France; 4 The George Institute for Global Health, Sydney, NSW 2042, Australia

**Keywords:** nutritional labelling, food choices, comprehension, perception, Dutch consumers, food policies

## Abstract

Front-of-pack labels (FoPLs) are efficient tools for helping consumers identify healthier food products. Although discussions on nutritional labelling are currently ongoing in Europe, few studies have compared the effectiveness of FoPLs in European countries, including the Netherlands. This study aimed to compare five FoPLs among Dutch participants (the Health Star Rating (HSR) system, Multiple Traffic Lights (MTL), Nutri-Score, Reference Intakes (RIs), and Warning symbols) in terms of perception and understanding of the labels and food choices. In 2019, 1032 Dutch consumers were recruited and asked to select one product from among a set of three foods with different nutritional profiles, and then rank the products within the sets according to their nutritional quality. These tasks were performed with no label and then with one of the five FoPLs on the package, depending on the randomization arm. Finally, participants were questioned on their perceptions regarding the label to which they were exposed. Regarding perceptions, all FoPLs were favorably perceived but with only marginal differences between FoPLs. While no significant difference across labels was observed for food choices, the Nutri-Score demonstrated the highest overall performance in helping consumers rank the products according to their nutritional quality.

## 1. Introduction

Front-of-pack labels (FoPLs) have been identified as a promising strategy to help consumers make healthier food choices at the point of purchase [1,2,3] and encourage manufacturers to improve the nutritional composition of their products [4,5]. Notably, the implementation of FoPLs has been recommended by the World Health Organization as a ‘best-buy’ measure to help prevent non-communicable diseases [6]. Given their potential to change consumer food choice architecture, by providing readily interpreted nutritional information, the provision of FoPLs has been identified as an effective nudging strategy [7]. However, the multiplicity of existing schemes, potentially in the same market, may increase confusion among consumers [8]. More specifically in the European Union (EU), according to the regulation, FoPLs may only be voluntary, meaning multiple schemes may coexist [9]. In this context, a request for harmonization at the EU level has prompted new discussions by the EU commission since 2018 to modify the existing regulation [10]. Similar political discussions pertaining to the objectives and principles of FoPLs have been included within a Codex Alimentarius e-working group, highlighting government interest in this area [11]. Some European countries have already implemented FoPLs as part of national nutrition prevention programs. Examples of these FoPLs include the Green Keyhole in the Nordic countries since the 1980s [12], the Multiple Traffic Lights (MTL) in the United Kingdom since 2004 [13], the Reference Intakes label (RIs) implemented in 2006 following a voluntary initiative from manufacturers [14], and more recently, the Nutri-Score in France since 2017, and then in Belgium and Spain in 2018 [15]. Other FoPLs have been proposed in recent years, including the Evolved Nutrition Label by a consortium of manufacturers [16], the nutritional circles label proposed by the leading association of the German food sector BLL (*Bund für Lebensmittelrecht und Lebensmittelkunde*), or the battery system proposed by the Italian government. These latter schemes have not been validated by scientific evidence. 

Discussions are still on-going in several European countries as to the most efficient FoPL for their population. In the Netherlands, the ‘Choices’ system was in place between 2006 and 2016. Initially developed by food manufacturers, and then endorsed by the government, this scheme was abandoned following a request from consumers, as it led to confusion as to the ranking of some foods [17]. Recently, the Dutch government announced the possible introduction of a new FoPL in the Netherlands, and noted that further research should be conducted to identify which labels would perform the best for Dutch consumers [18].

Studies investigating consumer responses to different types of FoPLs have explored various dimensions of intrinsic qualities, such as perceptions, understanding, and/or choice. In this context, Grunert et al. proposed a theoretical framework defining the different steps of FoPL use from perception to use in purchasing situations [19]. Although examining each of these elements provides a clearer picture of consumer reactions to different types of FoPLs, the relative contribution of each of these dimensions to help select an effective scheme varies and requires further investigation. Studies investigating perceptions suggest that FoPLs are generally favorably perceived in the population. However, while positive attitudes for a given system are likely to be required for a scheme to be efficient, there may be a discrepancy between consumer preferences and actual performance of the scheme. Indeed, consumers, and especially those with a higher educational level, tend to prefer schemes providing a larger amount of information, although they may not be able to process this information in purchasing situations where decisions are made in very short time frame [20,21,22,23]. Objective understanding, defined as the capacity of consumers to understand the information provided by the label in the way that is intended by its designers [19], is usually tested through ranking tasks, in which consumers are exposed to products displaying a FoPL on the pack and are required to rank their relative healthiness compared to a condition with no label. Studies tend to suggest that this type of measure may show a more contrasted performance across FoPLs, thereby providing a better discrimination across different schemes. Studies investigating consumer choices following exposure to FoPLs have shown contrasting results, depending in particular on the type of method that was used (choice task, virtual/experimental supermarket, or in-store study) [24,25,26,27,28,29,30,31,32,33,34,35,36]. Globally, the results of these studies suggest that the effect of FoPLs on consumer choices may be of low magnitude, as consumer purchases are guided by a host of influences, of which nutrition may only be one of several drivers, including price and promotion in particular. However, at the population level, such effects would lead to a substantial impact in terms of public health, contributing to the reduction of the nutrition-related disease burden [37].

The aim of the present study was to assess consumer responses to different FoPLs currently implemented in different countries in the world, in a Dutch sample using the methodology of the FOP-ICE study; an international experimental study comparing the effectiveness of various FoPLs in 12 countries [38]. The effectiveness of five front-of-pack nutrition labels corresponding to different types of FoPL formats—Health Star Rating system, Multiple Traffic Lights, Nutri-Score, Reference Intakes, and Warning symbols—was investigated through the three following dimensions: perception, objective understanding, and food choices.

## 2. Materials and Methods

### 2.1. Population Study and Individual Characteristics

Participants were recruited in the Netherlands by a web panel provider (Pureprofile), applying quotas for sex (50% women), age (one third in each of the following categories: 18–30 years, 31–50 years, over 51 years), and yearly household income (one third in each of the following categories: low (<13,962 €), medium (13,962 €–28,135 €), and high (>28,135 €)). In the online questionnaire, individuals were first asked to provide information on socio-demographic, lifestyle, and nutrition-related characteristics, including sex, age, monthly household income, educational level, involvement in grocery shopping, self-estimated diet quality, and self-estimated level of knowledge in nutrition. Individuals were also asked to declare the frequency purchasing the tested food categories (pizzas, cakes, and breakfast cereals, with response options as “always”, “often”, “sometimes”, and “never”). Those who responded “never” to at least two of the three food categories were ineligible to participate.

The protocol of the study (similar to the FOP-ICE study) was approved by the Institutional Review Board of the French Institute for Health and Medical Research (IRB Inserm n°17-404 and 17-404 bis) and the Curtin University Human Research Ethics Committee (approval reference: HRE2017-0760). At the beginning of the survey, participants were invited to give their electronic consent.

### 2.2. Stimuli and Front-of-Pack Nutrition Labels

Three food categories (pizzas, cakes, and breakfast cereals) were selected according to two criteria [38]: (1) commonly available in Dutch supermarkets, and (2) contain products with wide variability in nutritional quality. In each food category, a set of three products with distinct nutrient profiles (higher, medium, and lower nutritional quality) was created, allowing a ranking of products according to their nutritional quality. In order to avoid potential bias on product evaluation (e.g., familiarity, habit), mock packages representing a fictional brand (“Stofer”) were developed. 

**Figure 1:**
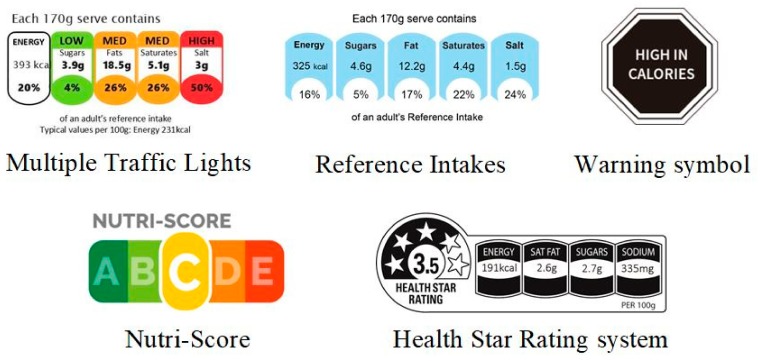
Front-of-pack nutrition labels tested in the present study.

Five FoPLs were tested in the present study (Figure 1), including both nutrient-specific and summary schemes. The nutrient-specific labels were: (1) the Multiple Traffic Lights (implemented in the United Kingdom in 2004), indicating the amounts of energy, fat, saturated fat, sugar, and salt, with a color (green, amber, red) depending on the amount; (2) the Reference Intakes, a monochromatic label displaying the amounts of the same nutrients; and (3) the Warning symbol (implemented in Chile in 2016), advising when the level of a given nutrient exceeds what is considered a healthy amount. Summary FoPLs included: (1) the Nutri-Score, a graded scale of five colors from dark green (associated with the letter A) to dark orange (associated with the letter E), characterizing the overall nutritional quality of the food or beverage and (2) the Health Star Rating system (implemented in Australia and New Zealand in 2014), using a graded scale of stars combined with information on nutrient amounts. 

### 2.3. Procedure

Participants were invited to respond to the online questionnaire that was presented in Dutch. Following the sociodemographic, lifestyle, and nutrition-related questions, participants were asked to complete the choice and understanding tasks, and then answer questions about their perceptions of the FoPL to which they had been assigned. 

**Figure 2:**
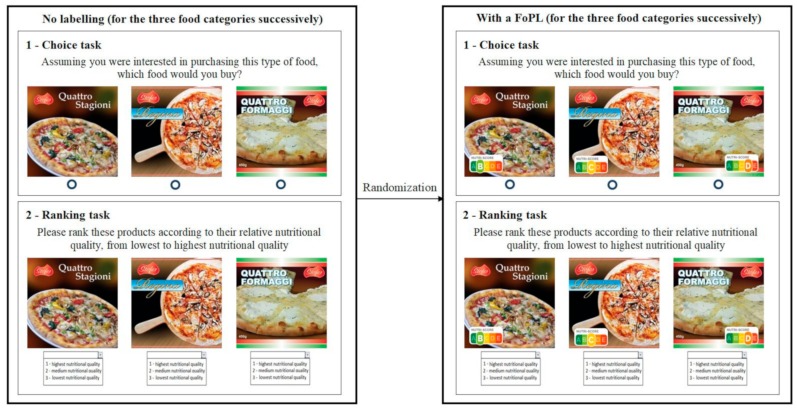
Procedure of the choice and ranking tasks for the pizza category.

Given that the first steps of the theoretical framework of FoPL use (perception and understanding) may influence the following step (food choices), the order of the dimensions was reversed in the experiment, starting with choice, followed by understanding and finally perception. First, for each food category, participants were asked to select the product they would be most likely to purchase without any FoPL shown on the mock packages. An “I wouldn’t buy any of these products” option was also available. After the choice task, participants were invited to rank the set of three products according to their nutritional quality (1—highest nutritional quality, 2—medium nutritional quality, and 3—lowest nutritional quality), with an “I don’t know” option also available and no FoPL on packages. Choice and ranking tasks were completed sequentially for the three food categories. Participants were then randomized to one of the five FoPLs and then invited to fulfill the same tasks, but this time with the assigned FoPL affixed to the mock packages. An example of the choice and ranking tasks for the pizza category is presented in Figure 2. 

Participants were then invited to respond to questions about their perceptions on the FoPLs. Various dimensions were assessed including liking (e.g., “I like this label”), awareness (e.g., “this label stands out”), and perceived cognitive workload (e.g., “this label is easy to understand”). For each question, participants provided their responses on a 9-point Likert scale ranging from “strongly disagree” to “strongly agree”.

### 2.4. Statistical Analyses

#### 2.4.1. Food Choice

For the choice analyses, +1 point was attributed when the lowest nutritional quality product was selected by the participant, +2 points for the medium nutritional quality product and +3 points for the highest nutritional quality product, first for the no labelling condition and then for the FoPL condition. Hence, for each food category, a score was computed using the difference of points between the two conditions, resulting in a discrete continuous score ranging from −2 to +2 points. A global score was finally calculated by summing the score of each category, resulting in a final score between −6 and +6 points. The percentage of participants who deteriorated or improved in their food choices between the no label and FoPL conditions was calculated for each FoPL group by food category. An ordinal logistic regression model was conducted to measure the association between the choice score and FoPL type. Only participants selecting a product in both the no label and FoPL conditions were included in the analyses. 

#### 2.4.2. Objective Understanding

Objective understanding of the FoPLs by consumers was assessed by the ability of participants to correctly rank the sets of products according to nutritional quality. A response was considered correct when the three products in the set were correctly ranked, leading to a +1 point score for the category. One error (or more) in the ranking task resulted in a −1 point score, while 0 points were attributed when participants selected the “I don’t know” answer. Thus, for each food category, a score for ranking ability was calculated using the difference in the number of points between the no label and FoPL conditions, ranging from −2 to +2 points, and leading to a global score of between −6 and +6 points for the three food categories combined. The percentage of correct answers in the no labelling and FoPL conditions was calculated by FoPL type and food category. An ordinal logistic regression model was performed to measure the association between the understanding score and FoPL type. 

For the choice and understanding analyses, models were adjusted for individual characteristics including sex, age, level of household monthly income, educational level, involvement in grocery shopping, self-estimated diet quality, and nutrition knowledge, and finally on the response to the question “did you see this label during the survey?”. The reference of the models for the FoPL categorical variable was the Reference Intakes label. Interactions between FoPLs and individual characteristics were tested, and stratified analyses were performed when the *p*-value of the interaction term was ≤0.10.

#### 2.4.3. Perception

The responses for the assessed perception aspects were characterized for each label by using means and standard deviations. To investigate the contribution of the different questions to the overall perception of FoPLs, principal component analysis was performed. Active variables were “this label is confusing”, “I like this label”, “this label does not stand out”, “this label is easy to understand”, “this label takes too long to understand”, “this label provides me the information I need”, and “I trust this label”. Dimensions, corresponding to a linear combination of active variables, have an eigenvalue reflecting the total variance explained by the dimension. The number of retained dimensions was chosen to obtain a cumulative percentage of acceptable variance. In the present study, only the first two dimensions were selected, simplifying the presentation. The contribution and coordinates of each active variable on each axis were computed, indicating how variables contribute to dimensions, and to what extent. The label was considered as a qualitative supplementary variable (not used to compute the dimensions, but mapped on the existing axes). Due to the combination of positive and negative framing of the perception questions, participants who provided the same answers to all perception questions were excluded from the analyses, except those consistently giving a score of five, which indicates a neutral perception. 

All analyses in the present study were conducted on SAS statistical software (PROC LOGISTIC, PROC PRINCOMP). Statistical tests were two-sided and a *p*-value ≤ 0.05 was considered statistically significant.

## 3. Results

### 3.1. Description of the Sample

Individual characteristics of the study sample are described in Table 1. The present study included 1032 Dutch participants, with 50% women, 33% over 51 years, 32% with a primary or secondary educational level, and 34% with a low household monthly income. Among all participants, 72% were responsible for grocery shopping, 11% had a very or mostly unhealthy diet quality, and 16% had no or little knowledge about nutrition.

### 3.2. Food Choices

The percentage of participants who modified their food choices between the no label and FoPL conditions is described in Figure S1. While within each food category and for all five FoPLs, a large number of participants did not change their choice between the two conditions (between 50% to 63% depending on the food category and the FoPL), or did not select any product (between 22% to 41% depending on the food category and the FoPL), significant modifications in choices occurred in the pizza and cake categories (overall *p*-value for the Bowker disagreement test = 0.0008 and 0.0001, respectively). Among participants who modified their food choices, a higher percentage demonstrated an improvement in the nutritional quality of their choices (between 2.9% and 10.7% depending on the label and the food category) compared to those demonstrating deterioration (between 2.9% and 5.8% depending on the label and the food category), with similar results found for the five individual labels.

Results of the associations between FoPLs and food choices are displayed in Table 2. Compared to the RIs, no significant association was found between FoPLs and the change in nutritional quality of food choices, overall and by food category, except for the Warning symbol. Exposure to the Warning symbol encouraged participants to select a healthier breakfast cereal.

### 3.3. Objective Understanding

The percentage of correct answers in the nutritional quality ranking task and the improvement between the no label and FoPL conditions are presented (according to FoPL type and food category) in Figure S2. Across all three food categories, the Nutri-Score produced the largest improvement in the percentage of correct answers compared to no label, followed by the MTL. For the other FoPLs, results differed depending on the food category. The associations between FoPL type and the ability to correctly rank products are presented in Table 3, with the RIs label as reference in the models. Overall, the Nutri-Score was the only FoPL to significantly improve participants’ ability to correctly rank products according to their nutritional quality compared to the RIs (odds ratio (OR) = 3.60 [2.48–5.24] (*p*-value < 0.0001)), while the other FoPLs did not show any significant results. Similar results were found for the three food categories, except for cakes where the Warning symbol (OR = 2.10 [1.32–3.34], *p*-value = 0.002) and MTL (OR = 1.66 [1.05–2.62], *p*-value = 0.03) also significantly improved the ranking ability of participants compared to the RIs, but Nutri-Score remained the label with the highest performance for cakes as well (OR = 4.52 [2.89–7.06], *p*-value < 0.0001). 

In sensitivity analyses where respondents who answered “I don’t know” were not included, similar trends were observed, though with even higher magnitudes of the effect of FoPLs (Table S1). 

No significant interaction with individual characteristics was found, except with sex. However, the interaction was quantitative.

### 3.4. Perception

The average scores for all perception questions are displayed in Figure S3. Overall, homogeneous results were observed between FoPLs on the various items that were investigated. From principal component analysis, two main dimensions were identified, explaining 44.8% and 21.1% of the total variance, respectively. The contribution values and coordinates of active variables on these two dimensions are displayed in Table 4. The first dimension (horizontal axis) was a linear combination of the responses to the following items: “this label is easy to understand” and “this label provides me the information I need” (which were positively associated with the first dimension), and “this label is confusing” and “this label takes too long to understand” (which were negatively associated with this dimension). The second dimension (vertical axis) was a linear combination of the responses to the following items: “this label takes too long to understand”, “this label does not stand out”, and “I like this label”, which were positively associated with this dimension. 

**Figure 3:**
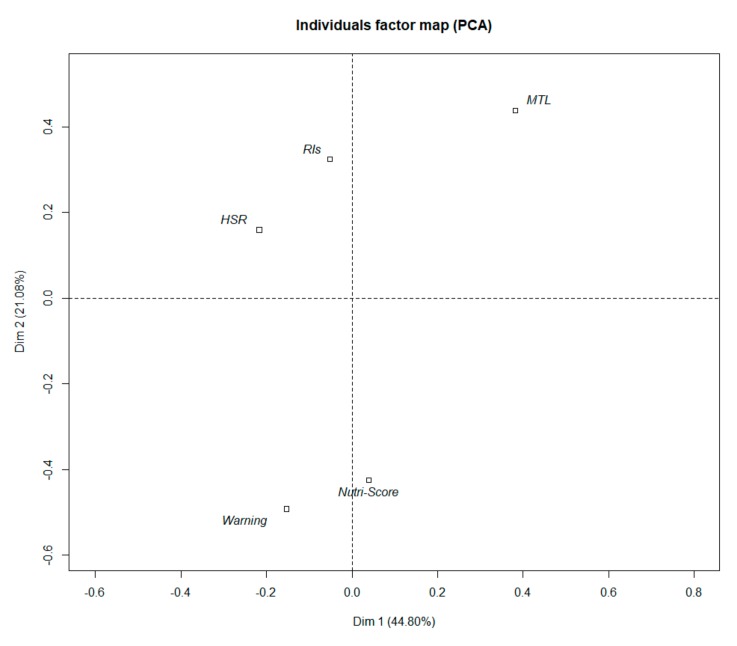
Principal component analysis map showing projection of the labels on the two axes.

When the label was mapped on the two axes as an illustrative variable, the graphic in Figure 3 was obtained. Differences between the FoPLs on the two dimensions appeared to be of very low magnitude (the position on the dimensions was between −0.5 and +0.5), although the MTL appeared opposed to the Nutri-Score and the Warning symbol on the second dimension. The MTL therefore appeared to somewhat be the preferred label, but compared to the Nutri-Score and Warning symbol, the MTL took too long to understand and did not stand out. 

## 4. Discussion

While no significant discrimination across FOPLs was observed in terms of perceptions and effect on food choices, the analyses of objective understanding of the labels showed significant differences across schemes. The Nutri-Score demonstrated the highest performance compared to the Reference Intakes in helping Dutch consumers identify and rank the nutritional quality of foods. The other FoPLs did not show any significant effects compared to the RIs except the MTL and Warning symbol for cake products, but to lesser extents. These results, specific to Dutch consumers, are consistent with the findings of the FOP-ICE study, where stronger overall performance of the Nutri-Score was observed for participants’ ability to correctly rank the nutritional quality of products in all countries, including the following European countries: Bulgaria, Denmark, France, Germany, Spain, and the United Kingdom [38,39,40].

The analyses exploring consumers’ perceptions of the FoPLs showed that all five FoPLs were favorably perceived. While variations across participants were substantial on the two dimensions of the principal component analysis, the differences by FoPL type were much smaller in magnitude. Moreover, familiarity appeared to influence perceptions, as RIs—that have been implemented as front-of-pack labels on the majority of food products worldwide since 2006—appeared to be appreciated by consumers compared to other labels. Finally, labels providing more accurate information (nutrient-based approaches with numerical information) appeared to be considered somewhat more trustworthy, especially among individuals with higher educational level or substantial knowledge, according to the literature, although they were less salient and entailed a higher cognitive workload [21,22,23,41]. The limited ability of studying perception to discriminate across labels might be related to the inter-subject approach used in this study (each participant was exposed to one FoPL only), while an intra-subject approach may have yielded more contrasted results (all participants exposed to all FoPLs).

Most previous studies investigating the effects of FoPLs on food choices have focused on the MTL or the RIs and their variants, and have yielded somewhat mixed results. The findings of these studies have typically shown that the RIs have no or limited effect on food choices [35,42,43,44], whereas the more interpretive MTL can help guide consumers towards healthier foods [26,28,33,35,45,46]. Few studies have investigated more recent schemes, including the Warning symbol, the HSR, and the Nutri-Score, and even fewer in a comparative design, though the results to date in studies using choice sets or experimental design in supermarkets have suggested that these interpretive labels can have a positive effect on the nutritional quality of food choices [30,32,35,42,44,47,48,49]. A recent study observed a significant improvement in the nutritional quality of food choices associated with the use of a warning label, while no results were observed for the other tested labels (MTL, HSR, and Nutri-Score); nevertheless larger sets (20 products) were used compared to our study, allowing capture of the differences for some labels [50]. Results of studies using choice sets, as in our study, appear to be influenced by the categories of products selected [49], as well as the size and types of products within the choice set [24]. When the effects of FoPLs were investigated in studies assessing purchasing outcomes, the Nutri-Score appeared to have a significant impact [30,32,47,48], while results were contrasted for other labels [27,34,47,51,52,53,54,55,56,57]. The non-significant effects observed on food choice in the present study could be related first to the use of mock packages featuring a fictional brand differing from a real world setting, and second to the type of methodology that was used. Indeed, even if the experimental design allowed control over potential confounding factors and other purchasing determinants, the choice tasks focused on three products from three food categories only, which limits the magnitude of the effects that could be observed compared to studies measuring the overall shopping cart. However, in our case the number of sets and products within the sets had to remain limited given that three dimensions were investigated in the same survey and the questionnaire could not be too long for participants to complete. In addition, choice and ranking tasks were performed on the same sets and included three products only. Indeed, the ranking of products according to nutritional quality had to be similar regardless of the FoPL used, and the higher the number of products within the set, the harder it is to achieve. The balance between the number of products for each task and overall simplicity for participants was carefully considered. Finally, the results could have been impacted by familiarity with and purchasing habits for the food categories used in the study. However, this bias was minimized first by the use of fictional products and a fictional brand, and second by the fact that participants who declared having never purchased one of the food categories were excluded from the analyses on that specific food category.

Our results on consumer understanding confirmed that interpretive systems, and in particular color-coded FoPLs, have greater potential than purely informative systems to improve the capacity of Dutch consumers to correctly rank the nutritional quality of foods. In our study, compared to the RIs, the Nutri-Score outperformed the other FoPLs in improving consumers’ ability to correctly rank products according to nutritional quality. These findings are consistent with the results of studies conducted in Uruguay [42,58], Australia [59], and other European countries [38,39,40,60]. Summary indicators have been demonstrated to be easier to understand by consumers [43,60,61], whereas nutrient-specific labels require greater cognitive workload. Color-coding, using in particular the green/red scale, provides an easy-to-interpret signal, associated with ‘stop’ and ‘go’ signals [62], and has been shown to increase attentional capture [58,63]. Moreover, from a biological perspective, red and green are immediately discerned and discriminated by the human eye [64]. Thus, a FoPL combining both summary and color-coded features, such as the Nutri-Score, is associated with a better objective understanding by consumers [38,60,65].

Another interesting issue raised by our results is the relative contribution of the different dimensions (and studies thereof) developed to characterize FoPLs and to compare the efficiency of different models. Overall, this study provides useful information on the relative contribution of each type of dimension to policy-makers in the selection of a FoPL. Consumers’ perceptions of FoPLs suggest that all types of labels are considered acceptable by consumers, with a limited discrimination across schemes, especially when using an inter-subject approach. Of greater concern is the finding of discrepancies between label preferences and performance, with the Nutri-Score displaying significantly higher performance on objective understanding compared to the other labels, while at the same time being perceived as less reliable by Dutch participants. By contrast, FoPLs considered more trustworthy and useful (RIs in particular), did not significantly improve the ability of participants to correctly rank the nutritional quality of products. This finding suggests that performance studies relying on the testing of consumer understanding may be one of the most important study types, allowing discrimination across label types, and therefore helping policy-makers in decision-making. Finally, results on choice suggest that FoPLs may yield limited effects on consumer choices, but that the results are highly dependent on the type of study that is performed, and in particular on the choice set and task consumers are asked to perform. Studies involving experimental conditions mimicking real-life purchases with a high number of choices and high variability in the nutritional quality of the foods offered may provide more contrasted results across labels and would be also one of the most important potential effects of FoPLs to investigate.

Strengths of our study include the participation of a large number of Dutch consumers from various sociodemographic groups, the investigation of multiple dimensions of FoPL effectiveness, and the comparison across multiple types of FoPL schemes using a randomized approach. A potential learning effect was also avoided by using a randomization of the presentation order within the sets and across food categories. Nevertheless, some limitations need to be acknowledged. First, Dutch participants were recruited online using set quotas, rather than attempting to generate a population representative sample, which requires caution regarding the extrapolation of the results. Moreover, although we were able to take into account several aspects of socio-cultural background, we did not include information on ethnicity, while it may affect consumer responses to FoPLs. Second, as participants were blinded to the hypotheses, no information was provided as to the objective or meaning of the FoPL to which they were exposed. While this reduced priming, it may have led to less favorable perceptions of less familiar FoPLs and to an underestimation of the labels’ effects. Moreover, participants did not have access to the nutritional composition of the products used in the study, which differs from real-life situations and might have led to fewer correct responses in the no label condition in the understanding task compared to what would occur in real life settings. However, this limitation applied equally to all FoPLs included in the study. Finally, participants were randomized to one FoPL, which led to an inter-subject comparison of the effects of FoPLs. Combining intra- and inter-subject approaches may yield more contrasted results across FoPLs, as shown in earlier studies [20,22,41,60].

To conclude, it is of major importance to investigate various dimensions of effectiveness before implementing a FoPL in a country; however, all dimensions do not necessarily have the ability to discriminate FoPL performance. It is important to note that even if a FoPL is favorably perceived and liked by consumers, it does not guarantee that it will be well understood and used to inform food choices. Thus, before selecting a FoPL, it appears essential to investigate consumers’ ability to understand and use various schemes, as this ability constitutes an essential step for a label to be effective in influencing food purchases and consumption. Among the different label types tested in the study, the Nutri-Score appears to be a valid alternative to help Dutch consumers identify and rank the nutritional quality of food products.

## Figures and Tables

**Table 1:** Individual characteristics of the study sample from Netherlands (N = 1032).

	N	%
**Sex**		
Men	517	50.1
Women	515	49.9
**Age, years**		
18–30	345	33.43
31–50	343	33.24
≥ 51	344	33.33
**Educational level**		
Primary education	13	1.26
Secondary education	314	30.43
Trade certificate	277	26.84
University, undergraduate degree	329	31.88
University postgraduate degree	99	9.59
**Level of household monthly income**		
High	342	33.14
Medium	343	33.24
Low	347	33.62
**Responsible for grocery shopping**		
Yes	746	72.29
No	55	5.33
Share job equally	231	22.38
**Self-estimated diet quality**		
I eat a very unhealthy diet	8	0.78
I eat a mostly unhealthy diet	102	9.88
I eat a mostly healthy diet	865	83.82
I eat a very healthy diet	57	5.52
**Nutrition knowledge**		
I do not know anything about nutrition	7	0.68
I am not very knowledgeable about nutrition	157	15.21
I am somewhat knowledgeable about nutrition	744	72.09
I am very knowledgeable about nutrition	124	12.02
**Did you see the FOP label during the survey?**		
No	293	28.39
Unsure	133	12.89
Yes	606	58.72
**Participants who recalled seeing the FoPL they were exposed to**		
*HSR*	111	53.62
*MTL*	135	65.53
*Nutri-Score*	147	71.36
*RIs label*	136	53.88
*Warning symbol*	77	37.20

HSR: Health Star Rating system; MTL: Multiple Traffic Lights; RIs: Reference Intakes.

**Table 2:** Associations between front-of-pack label type and change in nutritional quality of food choices by food category (N = 1032).

Food Category	N	HSR		MTL		Nutri-Score		Warning Symbol	
		OR (95% CI)	*p*	OR (95% CI)	*p*	OR (95% CI)	*p*	OR (95% CI)	*p*
All categories	898	1.21 [0.76–1.94]	0.4	0.94 [0.59–1.51]	0.8	1.10 [0.69–1.75]	0.7	1.32 [0.82–2.13]	0.3
Pizzas	692	1.11 [0.58–2.10]	0.8	0.85 [0.45–1.64]	0.6	0.76 [0.40–1.44]	0.4	0.88 [0.45–1.73]	0.7
Cakes	744	0.81 [0.44–1.49]	0.5	0.90 [0.50–1.63]	0.7	1.10 [0.61–1.98]	0.7	0.93 [0.50–1.71]	0.8
Breakfast cereals	643	1.72 [0.84–3.50]	0.1	0.93 [0.46–1.88]	0.8	1.77 [0.87–3.60]	0.1	2.99 [1.45–6.21]	0.003

The reference of the multivariate ordinal logistic regression for the categorical variable ‘label’ was the Reference Intakes. The multivariate model was adjusted for sex, age, educational level, level of income, responsibility for grocery shopping, self-estimated diet quality, self-estimated nutrition knowledge level, and “did you see this label during the online survey?” HSR: Health Star Rating system; MTL: Multiple Traffic Lights; OR: Odds Ratio; CI: Confidence Interval. Bold values correspond to significant results (*p*-value ≤ 0.05).

**Table 3:** Associations between FoPLs and the ability to correctly rank products according to nutritional quality by food category (N = 1032).

Food Category	N	HSR		MTL		Nutri-Score		Warning Symbol	
		OR (95% CI)	*p*	OR (95% CI)	*p*	OR (95% CI)	*p*	OR (95% CI)	*p*
All categories	1032	1.20 [0.82–1.75]	0.3	1.31 [0.90–1.90]	0.2	3.60 [2.48–5.24]	<0.0001	1.23 [0.84–1.81]	0.3
Pizzas	972	1.37 [0.85–2.21]	0.2	1.17 [0.73–1.88]	0.5	2.12 [1.34–3.37]	0.001	1.00 [0.62–1.62]	1.0
Cakes	1019	1.42 [0.89–2.24]	0.1	1.66 [1.05–2.62]	0.03	4.52 [2.89–7.06]	<0.0001	2.10 [1.32–3.34]	0.002
Breakfast cereals	931	0.90 [0.56–1.47]	0.7	1.00 [0.62–1.62]	1.0	2.66 [1.68–4.21]	<0.0001	0.85 [0.52–1.39]	0.5

The reference of the multivariate ordinal logistic regression for the categorical variable ‘label’ was the Reference Intakes. The multivariate model was adjusted for sex, age, educational level, level of income, responsibility for grocery shopping, self-estimated diet quality, self-estimated nutrition knowledge level, and “did you see this label during the online survey?” HSR: Health Star Rating system; MTL: Multiple Traffic Lights; OR: Odds Ratio; CI: Confidence Interval. Bold values correspond to significant results (*p*-value ≤ 0.05).

**Table 4:** Contributions and coordinates of active variables on the two dimensions from the principal component analysis.

Questions	Contributions		Coordinates	
	Dimension 1	Dimension 2	Dimension 1	Dimension 2
This label is confusing	19.59	12.88	−1.65	0.92
I like this label	10.40	18.14	1.20	1.09
This label does not stand out	7.09	20.36	−0.99	1.15
This label is easy to understand	18.51	2.03	1.61	0.36
This label takes too long to understand	15.06	22.64	−1.45	1.22
This label provides me the information I need	16.58	13.28	1.52	0.93
I trust this label	12.76	10.66	1.33	0.84
HSR	-	-	−0.22	0.16
MTL	-	-	0.38	0.44
Nutri-Score	-	-	0.04	−0.43
RIs label	-	-	−0.05	0.32
Warning symbol	-	-	−0.15	−0.49

Labels do not have contribution values given that they were considered as qualitative supplementary variables and were thus not used to compute the dimensions.

## Supplementary Materials

The following are available online at https://www.mdpi.com/2072-6643/11/8/1817/s1, Table S1: Associations between FoPLs and the ability to correctly rank products according to nutritional quality by food category: sensitivity analyses (N = 1032); Figure S1: Percentage of participants that deteriorated or improved their food choices between the two labelling situations, by food category and FoPL; Figure S2: Percentage of correct answers for the ranking tasks, by food category and FoPL; Figure S3: Average scores with standard deviation of perception questions by FoPL.
