# Supplementary material for: Consumers’ Responses to Front-of-Pack Nutrition Labelling: Results from a Sample from The Netherlands
Source: Nutrients. 2019 Aug 6;11(8):1817. doi: 10.3390/nu11081817 (PMC6723811; doi:10.3390/nu11081817)
Supplement: Supplementary file 1 [file nutrients-11-01817-s001.pdf]

Table S1. Associations between FoPLs and the ability to correctly rank products according to nutritional quality by food category: sensitivity analyses (N=1,032)

Food category	N	HSR		MTL		Nutri-Score		Warning symbol	
		**OR (95% CI)**	***p*P**	**OR (95% CI)**	***p*P**	**OR (95% CI)**	***p*P**	**OR (95% CI)**	***p*P**
All categories	1032	1.20 [0.82-1.75]	0.3	1.31 [0.90-1.90]	0.2	3.60 [2.48-5.24]	<.0001	1.23 [0.84-1.81]	0.3
Pizzas	972	1.37 [0.85-2.21]	0.2	1.17 [0.73-1.88]	0.5	2.12 [1.34-3.37]	0.001	1.00 [0.62-1.62]	1.0
Cakes	1019	1.42 [0.89-2.24]	0.1	1.66 [1.05-2.62]	0.03	4.52 [2.89-7.06]	<.0001	2.10 [1.32-3.34]	0.002
Breakfast cereals	931	0.90 [0.56-1.47]	0.7	1.00 [0.62-1.62]	1.0	2.66 [1.68-4.21]	<.0001	0.85 [0.52-1.39]	0.5

[a] The reference of the multivariate ordinal logistic regression for the categorical variable 'label' was the Reference Intakes.
The multivariate model was adjusted on sex, age, educational level, level of income, responsibility for grocery shopping, self-estimated diet quality, self-estimated nutrition knowledge level, and "Did you see this label during the online survey?"
The "I don't know" answers are not taken into account.
HSR: Health Star Rating system; MTL: Multiple Traffic Lights; OR: Odds Ratio; CI: Confidence Interval.
Bold values correspond to significant results (p-value≤0.05).

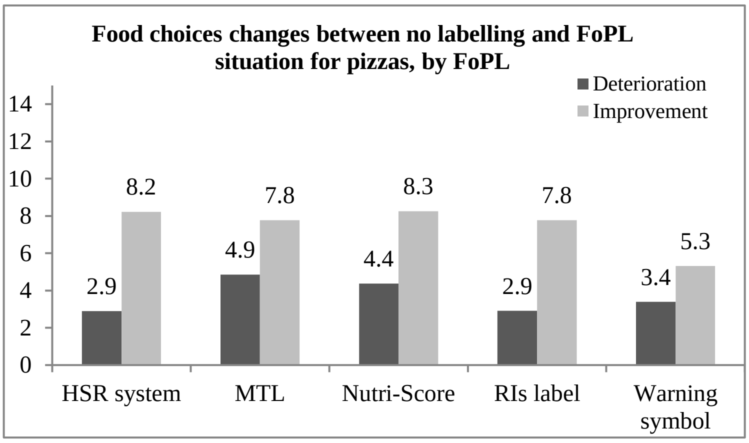

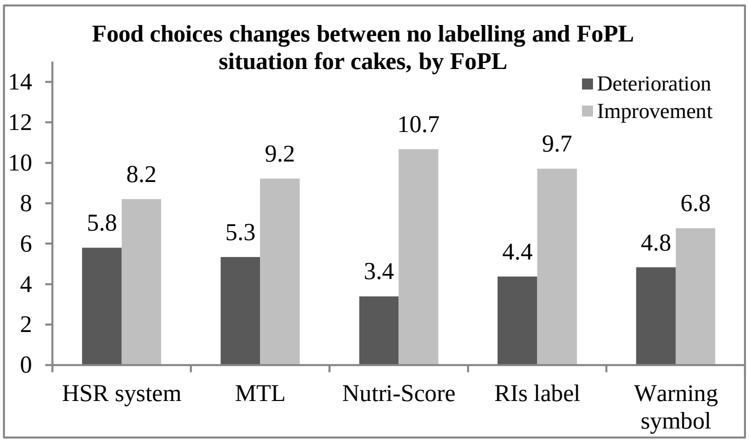

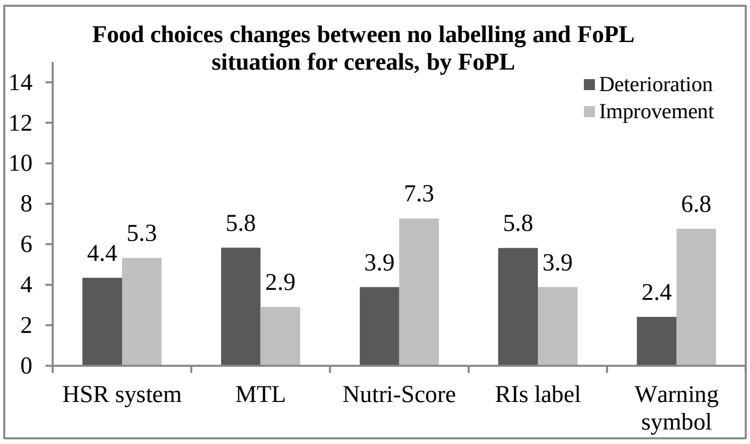

Figure S1. Percentage of participants having deteriorated or improved their food choices between the two labelling situations, by food category and FoPL

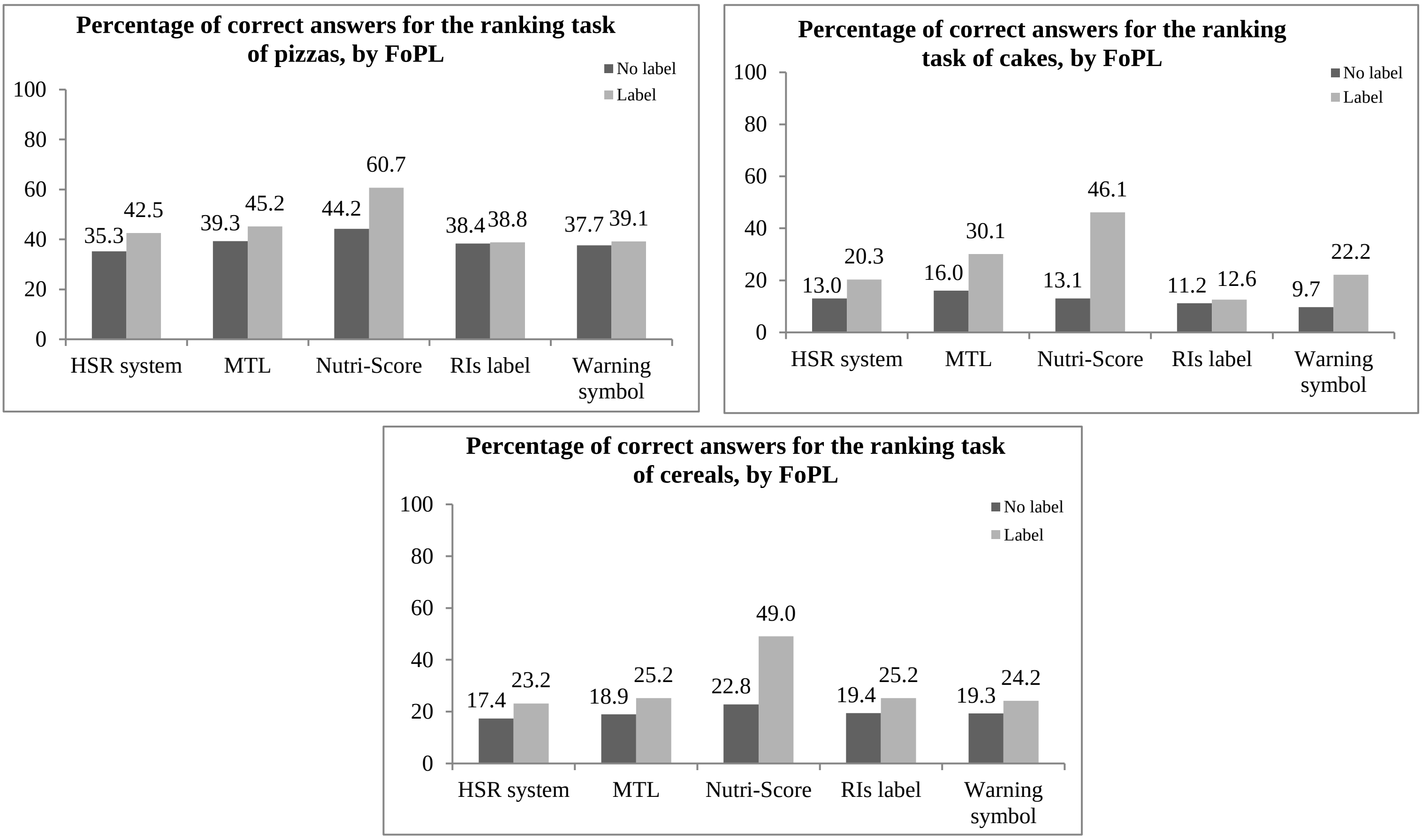

Figure S2. Percentage of correct answers for the ranking tasks, by food category and FoPL

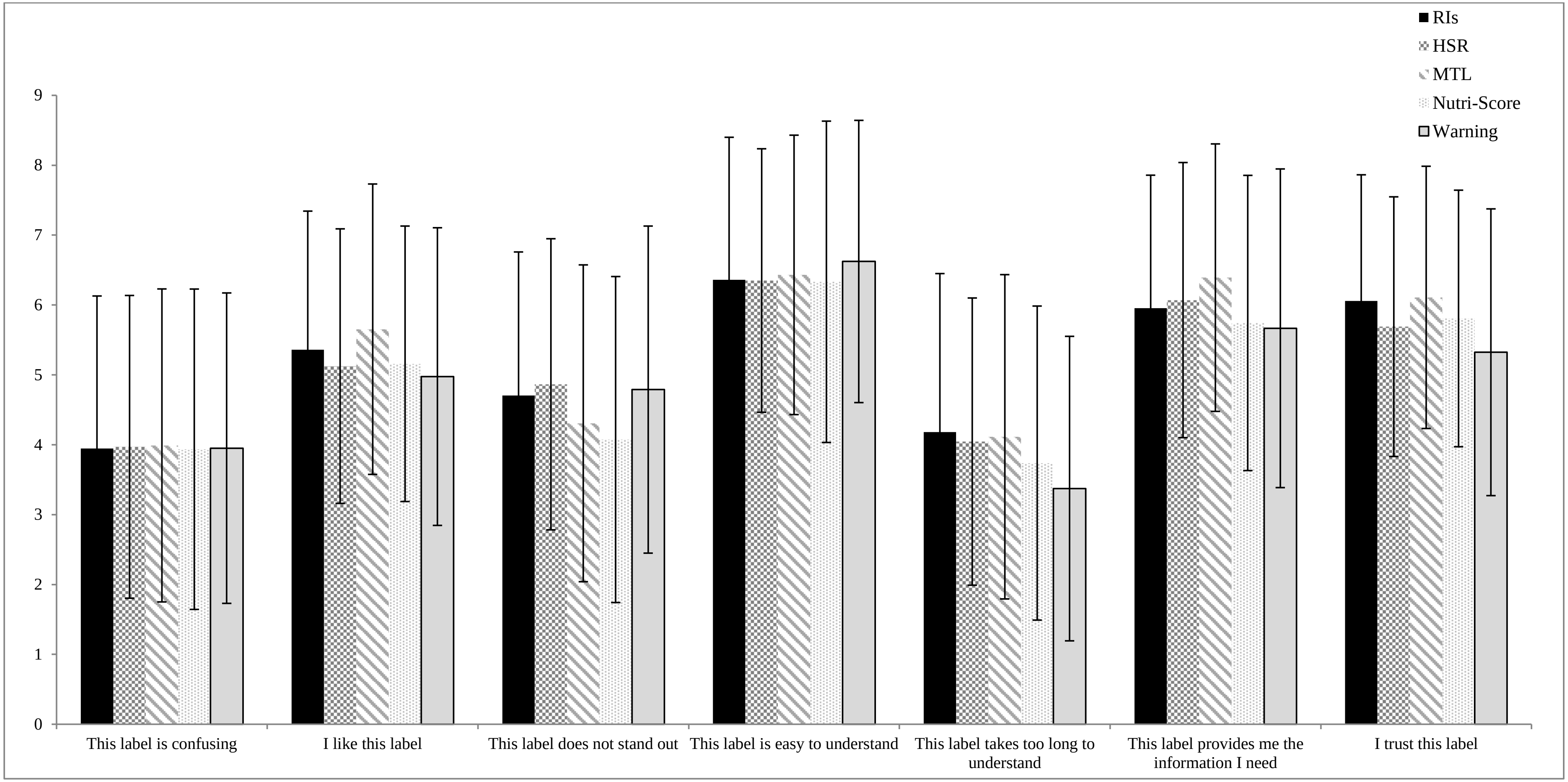

Figure S3. Averages scores with standard deviation of perception questions by FoPL
